# Brief International Cognitive Assessment for Multiple Sclerosis (BICAMS): discrete and regression-based norms for the Brazilian context

**DOI:** 10.1590/0004-282X-ANP-2020-0526

**Published:** 2021-11-30

**Authors:** Carina Tellaroli Spedo, Danilo de Assis Pereira, Seth Edward Frndak, Vanessa Daccach Marques, Amilton Antunes Barreira, Alan Smerbeck, Pedro Henrique Rodrigues da Silva, Ralph H. B. Benedict

**Affiliations:** 1 Universidade de São Paulo, Faculdade de Medicina de Ribeirão Preto, Hospital das Clínicas de Ribeirão Preto, Departamento de Neurociências e Ciências do Comportamento, Ribeirão Preto SP, Brazil. Universidade de São Paulo Faculdade de Medicina de Ribeirão Preto Hospital das Clínicas de Ribeirão Preto Ribeirão Preto SP Brazil; 2 State University of New York, School of Medicine and Biomedical Sciences, Departments of Neurology and Psychiatry, Buffalo, New York, United States. State University of New York School of Medicine and Biomedical Sciences Departments of Neurology and Psychiatry Buffalo New York United States; 3 Institute of Technology Rochester, Rochester, New York, United States. Institute of Technology Rochester Rochester New York United States; 4 Instituto Brasileiro de Neuropsicologia e Ciências Cognitivas, Brasília DF, Brazil. Instituto Brasileiro de Neuropsicologia e Ciências Cognitivas Brasília DF Brazil; 5 Universidade de São Paulo, Faculdade de Filosofia, Ciências e Letras de Ribeirão Preto, Departamento de Física, Laboratório Inbrain, Ribeirão Preto SP, Brazil. Universidade de São Paulo Faculdade de Filosofia, Ciências e Letras de Ribeirão Preto Departamento de Física Ribeirão Preto SP Brazil

**Keywords:** Multiple Sclerosis, Cognitive Dysfunction, Neuropsychology, Esclerose Múltipla, Disfunção Cognitiva, Neuropsicologia

## Abstract

**Background:**

The Brief International Cognitive Assessment for Multiple Sclerosis (BICAMS) has been recently developed as a brief, practical, and feasible tool for cognitive impairment in multiple sclerosis (MS).

**Objective:**

This study aimed to provide continuous and discrete normative values for the BICAMS in the Brazilian context.

**Methods:**

Normatization was achieved using six hundred and one healthy controls from the community assessed at five Brazilian geopolitical regions.

**Results:**

Mean raw scores, T scores, percentiles, and Z scores for each BICAMS measure are provided, stratified by age and educational level. Regression-based norms were provided by converting raw scores to scaled scores, which were regressed on age, gender, and education, yielding equations that can be used to calculate the predicted scores. Regression analyses revealed that age, gender, and education significantly influenced test results, as in previous studies.

**Conclusions:**

The normative data of the BICAMS to the Brazilian context presented good representativeness, improving its use in daily clinical practice.

## RESUMO

**Antecedentes:**

O BICAMS foi desenvolvido como uma ferramenta breve, prática e confiável para avaliar o comprometimento cognitivo na esclerose múltipla (EM).

**Objetivo:**

Neste estudo, objetivamos fornecer dados normativos para o BICAMS.

**Métodos:**

Normatização foi realizada com seiscentos e um controles saudáveis​​ da comunidade avaliados das cinco regiões geopolíticas brasileiras.

**Resultados:**

Escores brutos médios, escore T, percentil e escore Z para cada medida do BICAMS são fornecidos e estratificados por idade e nível educacional. Normas baseadas em regressão foram obtidas através da conversão dos pontos brutos em pontos ponderados, produzindo parâmetros de regressão que podem ser usados para calcular os escores preditos. As análises de regressão revelaram que idade, gênero e educação influenciaram significativamente nos resultados do teste, assim como em estudos prévios.

**Conclusão:**

Normas do BICAMS para o contexto brasileiro apresentaram boa representatividade, contribuindo para a utilização na prática clínica diária.

## INTRODUCTION

Over 40% of multiple sclerosis (MS) patients can present cognitive impairment (CI)^1^^-^^4^. CI often manifests in decreased processing speed, learning and memory deficits, and less frequently in executive dysfunction^2^^,^^3^. Cognitively disabled patients are more likely to be unemployed and report fewer extra-curricular and social activities^5^^,^^6^. Appropriate test measures for the identification of CI are essential for the clinical management of the disease. Ideally, all MS patients would be routinely evaluated and/or monitored for CI, with similar measures being employed across specialized care centers. 

An extensive neuropsychological assessment can be costly and time-consuming, with standards for the cognitive evaluation varying between providers. The Brief International Cognitive Assessment for Multiple Sclerosis (BICAMS) is a collection of tests chosen by an international panel to standardize and facilitate routine monitoring of cognition in MS patients^7^. Transcultural studies between BICAMS from Brazil and the United States (US) have demonstrated its reliability and validity^8^. 

The present study was conducted to establish the discrete and regression-based norms of the BICAMS to the Brazilian context.

## METHODS

### Participants

Six hundred and one healthy control volunteers were recruited. The healthy controls were from the five Brazilian regions (north, northeast, south, southeast, and central-west), as recommended by the Brazilian Institute of Geographic and Statistics^9^. The Ethics Committee of the Medical School of Ribeirão Preto, University of São Paulo, approved all research procedures for this study. 

### Quality control of data acquisition

Qualification procedures were required from all the examiners. Psychologists and health professionals participated in an interactive training on administration and scoring procedures of the BICAMS. The protocol was administered in a standardized manner following commonly used manual-based instructions^10^. The two sub-scales of the Hospital Anxiety and Depression Scale (HADS)^11^ were also administered as markers of major depression and generalized anxiety disorder. The Mini-Mental State Examination test (MMSE)^12^ was administered for screening and to exclude cognitive impairment and possible dementia. No healthy controls were excluded based on their HADS or MMSE performance.

### BICAMS standardization and scoring procedures

 In brief, the Symbol Digit Modalities Test (SDMT)^13^ assesses complex scanning, visual tracking^14^, processing speed, and attention. The SDMT oral version was employed in this study, following previous validation studies^4^^,^^15^ and consensus opinion papers^16^. The task consists of a series of symbols presented at random, each with a blank space below, and participants are asked to name the number that matches each symbol. The maximum duration of the test is 90 seconds for the participant to complete each assessment (oral and written). When both forms of the test are administered, it is recommended that the written version be given first. It takes about five minutes to complete the test. The number of correct substitutions within the 90-second interval is recorded, with 110 being the maximum score for each form (written and oral). The translated standardized instructions of the SDMT for the Brazilian context are provided in the Technical and Interpretative Manual.

The California Verbal Learning Test -Second Edition (CVLT-2)^17^ is a commonly used test of auditory/verbal learning and memory^18^^,^^19^. The total CVLT-2 raw score is obtained by the sum of the correct words said in the five learning trials (T1 to T5). The total raw score for the learning trial obtained for the CVLT-2 is 80 points. The translated standardized instructions of the CVLT-2 to the Brazilian context are: *“Eu vou ler uma lista de palavras para você. Preste atenção porque quando eu terminar de ler eu quero que você me diga o máximo de palavras que puder. Você pode repeti-las em qualquer ordem me dizendo o máximo de palavras que você se lembrar. Você está pronto?* Leia a lista (A) de palavras em um mesmo ritmo, utilizando um pouco mais do que 1 segundo de intervalo entre as palavras. Dessa forma, a lista completa deve levar entre 18 e 20 segundos. E então diga: *Pode começar* **.”**

Instructions for the trials 2 to 5 (T2 to T5) in Brazilian Portuguese are: *“Eu vou ler para você novamente a mesma lista de palavras. Como da primeira vez, eu quero que você me diga o máximo de palavras que puder se lembrar e em qualquer ordem. Procure repetir as mesmas palavras que você me falou na primeira vez”.*

The Brief Visual Memory Test-Revised (BVMT-R)^20^ is a visual/spatial memory test wherein subjects view a stimulus card with six figures for 10 seconds, and then render the figures from memory. There are three learning trials, which are summed to produce the final BVMT-R score (maximum score 36). The scores reflect visuospatial memory. The translated standardized instructions of the BVMT-R to the Brazilian context are: *“Agora eu vou mostrar para você uma página com alguns desenhos. Eu preciso que você olhe atentamente para cada um deles durante 10 segundos. Procure prestar atenção no formato e na posição deles na folha. Eu vou te mostrar uma vez, daí retiro os desenhos e você os fará de memória aqui nesta folha (mostrar uma folha de sulfite A4 em branco). Nós faremos um treino, ou seja, vou te mostrar os desenhos e você irá desenhá-los por três vezes para você poder aprender e memorizar os desenhos.”*

Instructions for the trials 2 and 3 (T2 and T3) in Brazilian Portuguese are: *“Muito bem, agora vou mostrar mais uma vez porque quero ver se você aprendeu mais alguma figura”.* Present the page with the stimuli for 10 seconds, then remove and deliver the blank sheet.

### Statistical analysis

Statistical analysis was performed with the Statistical Package for the Social Sciences (v21.0, SPSS Inc, Chicago). Normality tests for each variable were performed with the Shapiro-Wilk test.

To the regression-based norms, the same methodology employed previously^21^^,^^22^ were applied in the present study. Each raw value was rank-ordered, converted to a proportion estimated, and then multiplied by 100 to derive percentile ranks. The ranks were then converted to scaled scores using the same conversion for all variables (mean=10, standard deviation=3). Thus, these steps allowed to convert the raw scores to a scaled score metrics based on a cumulative frequency distribution. The new variables are the Scaled Scores (SS) that were carried forward to the next step. 

The SSs were inspected to ensure if the data were normally distributed. The scaled scores were then entered one at a time, as the dependent variables in multiple regression analyses used to model the proportion of variance in performance accounted for by the block entry of four demographic variables: age, age², gender, and years of education. Age and age² were coded in years. The b weights for each demographic variable, a predictive constant, and the standard deviation of the group’s raw residuals were derived for each test.

## RESULTS

Demographics of the normative sample are shown in Table 1. The sample consisted of 65.6% (n=394) females, and age between 18 to ≥ 70 years. Most participants were between 30 and 39 years old (30.1%, n=181) and had a high educational level (59.6%, n=358, >12 years). The MMSE and HADS scores were classified as normal. 

**Table 1.** Demographic characteristics of the normative sample (n=601).

Demographic data		Percentage (freq.)	Mean±SD (95%CI)
Gender	Female	65.6 (394)	-
	Male	34.4 (207)	-
Region	Central West	32.1 (193)	-
	Northeast	6.5 (39)	-
	South	35.3 (212)	-
	Southeast	26.1 (157)	-
Age groups (years)	18 to 29	22.3 (134)	-
	30 to 39	30.1 (181)	-
	40 to 49	17 (102)	-
	50 to 59	17.5 (105)	-
	60 to 69	53 (8.8)	-
	≥70	26 (4.3)	-
Classes of education (years)	1 to 8	10.8 (65)	-
	9 to 11	29.6 (178)	-
	>12	59.6 (358)	-
MMSE		-	28.46±3.55 (13 to 30)
HADS-A		-	6.27±3.92 (0 to 21)
HADS-D		-	4.85±2.05 (0 to 19)

SD: standard deviation; IC: confidence interval; freq.: frequency; MMSE: Mini Mental State Examination; HADS-A: Hospital Anxiety and Depression Scale, anxiety measure; HADS-D: Hospital Anxiety and Depression Scale, depression measure.

In the present paper, the discrete and regression-based norms are provided, either of which can be used by the examiner.

### Using the discrete norms

The discrete norms, stratified by age and educational level, are shown in Tables 2 and 3.

**Table 2.** Discrete norms: BICAMS scores stratified by age and educational level.

Age	BICAMS tests	Educational level									
		1-11 years					>12 years				
		Mean	±SD	Percentile			Mean	±SD	Percentile		
				5th	10th	50th			5th	10th	50th
18-29 (n=134)	CVLT-II T1	8.1	2.8	4	4	8	7.7	2.2	5	5	7
	CVLT-II T2	10.4	2.6	5	6	11	10.6	2.3	7	8	10
	CVLT-II T3	12.2	2.3	8	8	13	12.4	2.5	8	9	13
	CVLT-II T4	12.8	2.5	7	9	13	13.4	2.5	10	11	14
	CVLT-II T5	13.4	2.2	8	10	14	14.1	2.2	10	11	15
	CVLT-II Total	56.8	10	36	40	57	58.2	9.3	41	45	59
	CVLT-II Total Rep.	3	4.7	-	-	-	3.3	3.7	-	-	-
	CVLT-II Total Intr.	1.5	2.8	-	-	-	1.5	4.1	-	-	-
	BVMT-R T1	7.4	2.9	2	3	8	7.3	2.9	2	3	7
	BVMT-R T2	9.3	2.4	4	6	10	9.5	2.2	4	7	10
	BVMT-R T3	10.3	2.3	4	7	12	10.6	1.8	6	8	11
	BVMT-R Total	27	7	10	17	27	27.5	5.9	15	19	28
	Oral SDMT	55	14	32	39	52	60	15.7	36	45	57
30-39 (n=181)	CVLT-II T1	7.6	2.4	4	5	8	7.4	2.2	4	5	7
	CVLT-II T2	10.2	2.4	6	7	10	10.6	2.6	6	7	10
	CVLT-II T3	11.7	2.4	8	8	12	12.4	2.8	6	9	13
	CVLT-II T4	12.3	2.4	8	9	12	13.1	2.4	9	10	13
	CVLT-II T5	12.9	2.1	10	10	13	13.9	2.2	9	11	15
	CVLT-II Total	54.8	9.1	35	39	53	57.4	10.2	38	44	58
	CVLT-II Total Rep.	5.6	5.4	-	-	-	4.8	5.9	-	-	-
	CVLT-II Total Intr.	1.1	1.8	-	-	-	1.3	2.1	-	-	-
	BVMT-R T1	6.1	3.6	2	2	5	6.7	2.9	2	3	6
	BVMT-R T2	8.4	3	3	4	8	9.3	2.4	5	6	10
	BVMT-R T3	10	2.5	5	6	11	10.5	1.9	6	8	11
	BVMT-R Total	24.4	8.1	10	13	26	26.6	6.3	15	19	27
	Oral SDMT	48.8	15	23	31	49	60.6	15.6	33	40	60
40-49 (n=102)	CVLT-II T1	6.5	2	4	4	6	7	2.2	4	5	7
	CVLT-II T2	9.8	2	7	7	10	9.9	2.6	5	6	10
	CVLT-II T3	11	2	7	8	11	11.7	2.4	8	9	11
	CVLT-II T4	11.9	2.5	5	9	12	12.8	2.3	8	10	13
	CVLT-II T5	12.9	1.8	10	10	13	13.2	2.3	9	10	14
	CVLT-II Total	52	8.3	35	30	51	54.5	9.3	39	42	55
	CVLT-II Total Rep.	5.2	3.7	-	-	-	5.4	6.4	-	-	-
	CVLT-II Total Intr.	1.1	1.3	-		-	1.2	1.9	-	-	-
	BVMT-R T1	5.6	3.3	0	1	5	5.7	2.5	2	2	6
	BVMT-R T2	8	2.9	2	4	9	8.4	2.7	3	5	9
	BVMT-R T3	9.1	31	1	4	11	10.1	2.4	4	6	11
	BVMT-R Total	22.8	8.4	6	12	23	24.2	6.6	10	13	26
	Oral SDMT	39.2	14.8	14	27	45	55.3	15.4	31	36	56

±SD: standard deviation; n: sample size; CVLT-II T1: California Verbal Learning Test second edition, trial 1; CVLT-II T2: California Verbal Learning Test second edition, trial 2; CVLT-II T3: California Verbal Learning Test second edition, trial 3; CVLT-II T4: California Verbal Learning Test second edition, trial 4; CVLT-II T5: California Verbal Learning Test second edition, trial 5; CVLT-II Total: California Verbal Learning Test second edition, Total sum score; CVLT-II Total Rep.: California Verbal Learning Test second edition, Total of Repetitions; CVLT-II Total Intr.: California Verbal Learning Test second edition, Total of Intrusions; BVMT-R T1: Brief Visuospatial Memory Test Revised, Trial 1; BVMT-R T2: Brief Visuospatial Memory Test Revised, Trial 2; BVMT-R T3: Brief Visuospatial Memory Test Revised, Trial 3; BVMT-R Total: Brief Visuospatial Memory Test Revised, Total Sum Score; Oral SDMT: Symbol Digit Modalities Test oral form.

**Table 3.** Discrete norms: BICAMS scores stratified by age and educational level (continuation).

Age	BICAMS tests	Educational Level									
		1-11 years					>12 years				
		Mean	±SD	Percentile			Mean	±SD	Percentile		
				5th	10th	50th			5th	10th	50th
50-59 (n=105)	CVLT-II T1	7.1	2.2	4	5	7	6.9	2.4	3	4	7
	CVLT-II T2	9	2.4	5	6	9	9.3	2.4	6	6	9
	CVLT-II T3	10.6	2.4	7	8	10	11	2.6	6	7	12
	CVLT-II T4	11	2.7	5	7	12	12	2.3	6	9	12
	CVLT-II T5	12.3	2.7	8	9	13	13.4	2.3	9	10	14
	CVLT-II Total	50	9.6	35	32	49	52.5	8.3	38	42	53
	CVLT-II Total Rep.	4.9	4.4	-	-	-	4,1	3.7	-	-	-
	CVLT-II Total Intr.	1.4	2	-	-	-	1.3	2.3	-	-	-
	BVMT-R T1	3.5	2.3	0	1	4	5.4	3.0	0	1	5
	BVMT-R T2	5.4	3.2	0	1	5	7.3	2.8	3	4	7
	BVMT-R T3	7	3.3	2	2	8	9.3	2.4	3	5	10
	BVMT-R Total	16	8	4	6	18	22	7.3	8	13	24
	Oral SDMT	36.9	14	12	20	33	46.4	14.9	26	28	47
60-69 (n=53)	CVLT-II T1	6.2	2.1	1	4	6	6	1.7	3	3	6
	CVLT-II T2	8.5	2.7	2	6	9	8.7	1.6	6	6	9
	CVLT-II T3	9.8	2.8	2	7	10	10.4	2.4	5	6	11
	CVLT-II T4	10.3	3.4	2	7	11	12	2.2	8	8	12
	CVLT-II T5	10.9	3.3	1	6	12	13.2	2.2	8	10	13
	CVLT-II Total	45.6	12.9	30	31	48	50.2	7.9	32	37	52
	CVLT-II Total Rep.	6	3.2	-	-	-	6.3	6.2	-	-	-
	CVLT-II Total Intr.	1.5	2.2	-	-	-	1.6	1.7	-	-	-
	BVMT-R T1	3.8	2.7	0	1	3	4.2	3.4	1	2	4
	BVMT-R T2	5.5	2.8	1	2	6	6.5	3.5	1	3	6
	BVMT-R T3	6.5	3.7	0	1	7	8.3	2.8	1	5	8
	BVMT-R Total	15.8	8.4	1	5	16	22	9.1	2	8	17
	Oral SDMT	29.5	12.9	10	12	30	44	14.4	23	26	40
>70 (n=26)	CVLT-II T1	5.2	2.0	1	3	5	6.6	1.8	5	5	6
	CVLT-II T2	7.5	2.2	4	4	7	8.1	2.3	4	4	9
	CVLT-II T3	8.7	2.3	5	5	9	8.4	1.6	6	6	8
	CVLT-II T4	9.8	2.6	6	6	10	9.7	2.2	6	6	10
	CVLT-II T5	10.3	2.7	5	6	10	12.1	2.5	8	8	13
	CVLT-II Total	41.5	9.7	21	30	42	45	8,2	29	29	47
	CVLT-II Total Rep.	6.0	4.6	-	-	-	5	4.6	-	-	-
	CVLT-II Total Intr.	1.1	1.7	-	-	-	3.4	4.7	-	-	-
	BVMT-R T1	2.5	2.5	1	1	2	6	3.7	1	1	5
	BVMT-R T2	3.9	3.2	1	1	3	7.9	3.7	2	2	7
	BVMT-R T3	4.6	3.6	1	1	4	8.4	3.7	2	2	9
	BVMT-R Total	11.1	8.5	1	1	10	19	10.7	5	5	20
	Oral SDMT	23.7	9.8	9	9	26	40.1	8.6	23	26	37

±SD: standard deviation; n: sample size; CVLT-II T1: California Verbal Learning Test second edition, trial 1; CVLT-II T2: California Verbal Learning Test second edition, trial 2; CVLT-II T3: California Verbal Learning Test second edition, trial 3; CVLT-II T4: California Verbal Learning Test second edition, trial 4; CVLT-II T5: California Verbal Learning Test second edition, trial 5; CVLT-II Total: California Verbal Learning Test second edition, Total sum score; CVLT-II Total Rep.: California Verbal Learning Test second edition, Total of Repetitions; CVLT-II Total Intr.: California Verbal Learning Test second edition, Total of Intrusions; BVMT-R T1: Brief Visuospatial Memory Test Revised, Trial 1; BVMT-R T2: Brief Visuospatial Memory Test Revised, Trial 2; BVMT-R T3: Brief Visuospatial Memory Test Revised, Trial 3; BVMT-R Total: Brief Visuospatial Memory Test Revised, Total Sum Score; Oral SDMT: Symbol Digit Modalities Test oral form.

### Using the regression-based norms

- Step 1: To obtain the predicted scores from raw scores, the examiner must convert the raw scores to scaled scores derived from the healthy controls using the data provided in Table 4.

**Table 4.** Regression-based norms: BICAMS raw-to-scaled scores conversions.

Scaled scores	Raw Scores			Classification
	CVLT-II	BVMT-R	SDMT	
1	<20			Extremely low
2	20-28			
3	29-31	1-2	1-9	
4	32-35	3-5	10-17	
5	36-39	6-8	18-23	Borderline
6	40-41	9-12	24-29	
7	42-44	13-17	30-36	Low average
8	45-48	18-20	37-43	
9	49-52	21-23	44-49	Average
10	53-56	24-26	50-53	
11	57-60	27-28	54-58	
12	61-64	29-30	59-62	High average
13	65-66	31-32	63-68	
14	67-69	33-34	69-74	Superior
15	70-71	35	75-79	
16	72	36	80-93	Very superior
17	73-74		94-107	
18	75		>107	
19	>75			

BICAMS: Brief International Cognitive Assessment for Multiple Sclerosis; SDMT: Symbol Digit Modalities Test; CVLT-II: California Verbal Learning Test second edition; BVMT-R: Brief Visuospatial Memory Test Revised.

- Step 2: To obtain the predicted scaled score (pss), the examiner must employ the predictive equation model. It is necessary to use the formula below (in gender, please use 1 for male and 2 for female) and the data from Table 5 (final regression models and standard deviation of the residual for BICAMS measures).

**Table 5:** Final regression models and standard deviation of residuals for the BICAMS measures.

CVLT2 Residual SD		BVMT-R Residual SD		SDMT Residual SD	
	2.527166		2.626665		2.48323
CVLT-2 Reg Model		BVMTR Reg Model		SDMT Reg Model	
Predictor	B	Predictor	B	Predictor	B
(constant)	8.512324	(constant)	11.58455	(constant)	9.248778
Age	-0.14798	age	-0.14752	age	-0.01094
age^2^	0.001373	age^2^	0.000896	age^2^	-0.00086
Sex	0.176426	sex	-0.19042	sex	-0.4714
education	0.364315	education	0.22895	education	0.263055

BICAMS: Brief International Cognitive Assessment for Multiple Sclerosis; SD: standard derivation; CVLT-2: California Verbal Learning Test second edition; SDMT: Symbol Digit Modalities Test; BVMT-R: Brief Visuospatial Memory Test Revised; Reg. Model: Regression Model.

Predicted Scaled Score (pss) = Constant + β _age_ (age) + β _age²_ (age^2^) + β _gender_ (gender) + β _ed._ (ed.)

- Step 3: In sequence, the examiner must calculate the Z-score using the formula:

Z-score = (Actual scaled score -Predicted scaled score) / SD of residuals (provided in Table 5).

## DISCUSSION

The present study aimed to provide both discrete and regression-based norms for the BICAMS. This study followed the recommendations of the international validation protocol for the BICAMS^10^^,^^16^ and is the first to publish in the Brazilian Portuguese the discrete and regression-based norms for BICAMS with age, gender and education corrections in the Brazilian population. 

The BICAMS is a validated and reliable brief protocol for monitoring the CI of MS patients. In several countries, efforts are being made to establish it as a psychometrically valid and reliable tool that is internationally applicable. This protocol was optimized for small MS centers so it can be administered by health care professionals without specific expertise in neuropsychological testing and without high costs. Its clinical use allows large-scale international clinical trials to have a common outcome measure of cognitive functioning^23^^,^^24^. It is recog nized that assessments and follow-ups of cognition should be as much as a priority as the evaluation of physical disability.

Discrete norms have their limitations and have come under criticism over in recent years, but they facilitate clinical use. Discrete norms are easier to use, but may distort demographic variables such as age, gender, and educational level, so they should be used with caution. Regression-based norms allow control of demographic influences on test performance. 

In conclusion, our results provide the normative data of the BICAMS for use in the Brazilian context. Future studies are necessary to confirm its suitability for longitudinal assessments. 
